# Visual electrophysiology in carotid endarterectomy: a systematic review

**DOI:** 10.1186/s40942-026-00895-2

**Published:** 2026-06-29

**Authors:** Sofia Pacheco-Moreira, João Rocha-Neves, Ana Marta, João Miguel Coelho, João Pinheiro-Costa, José P. Andrade, André Ferreira

**Affiliations:** 1 Faculty of Medicine, University of Porto, Al. Prof. Hernâni Monteiro, 4200-319 Porto, Portugal; 2 Department of Biomedicine - Unit of Anatomy, Faculty of Medicine of University of Porto, Porto, Portugal; 3 RISE-Health, Faculdade de Medicina, Universidade do Porto, Alameda Prof. Hernâni Monteiro, Porto, 4200-319 Portugal; 4 Department of Vascular Surgery, Unidade Local de Saúde – Alto Ave, Guimarães, Portugal; 5 Department of Ophthalmology, Neurosciences Department, Centro Hospitalar Universitário de Santo António, Unidade Local de Saúde de Santo António, Porto, Portugal; 6 Unit for Multidisciplinary Research in Biomedicine, School of Medicine and Biomedical Sciences, University of Porto (ICBAS), Porto, Portugal; 7 Department of Ophthalmology, ICBAS - School of Medicine and Biomedical Sciences, University of Porto, Porto, Portugal; 8 Unit of Ophthalmology, Instituto CUF, Porto, Portugal; 9 CINTESIS - Center for Health Technology and Services Research, Porto, Portugal

**Keywords:** Carotid stenosis, Carotid artery disease, Electroretinography, Visual evoked potentials, Visual electrophysiology, Retinal ischemia

## Abstract

**Background:**

To systematically evaluate changes in ocular electrophysiological function following carotid endarterectomy (CEA) and to determine whether electroretinography (ERG) and visual evoked potentials (VEP) can detect postoperative functional recovery of the retina and visual pathways.

**Methods:**

A systematic search of MEDLINE, Scopus, and Web of Science was conducted from inception to August 2025. Observational studies assessing electrophysiological outcomes (ERG, VEP, multifocal recordings, or electrooculography [EOG]) before and after CEA in adults with carotid stenosis were included. Study quality was evaluated using the NHLBI Study Quality Assessment Tools. Due to substantial clinical and methodological heterogeneity, a quantitative synthesis was not performed.

**Results:**

Four prospective observational studies were included, involving 95 patients with predominantly severe carotid stenosis (≥ 70%), with one cohort applying a ≥ 60% threshold. Electrophysiological findings were heterogeneous: VEP parameters generally showed no consistent postoperative improvement, whereas ERG demonstrated significant enhancement of oscillatory potentials and a-/b-waves amplitudes in 3 of the 4 included studies. Visual field indices improved in several cohorts, while structural parameters, particularly retinal nerve fiber layer thickness, remained stable. Changes in intraocular pressure were inconsistently reported. Overall methodological quality indicated a moderate-to-high risk of bias, primarily due to small sample sizes, lack of adjustment for confounders, and variability in electrophysiological protocols.

**Conclusion:**

The available evidence, while preliminary and exploratory, suggests a possible association between CEA and postoperative changes in electrophysiological parameters, most notably ERG-derived measures. These findings should be interpreted with considerable caution given the small number of studies, limited sample sizes, and substantial methodological heterogeneity. Whether ocular electrophysiology holds clinical utility in the perioperative assessment of carotid disease remains to be established by larger, adequately powered, and standardized prospective studies.

**Supplementary Information:**

The online version contains supplementary material available at https://doi.org/10.1186/s40942-026-00895-2.

## Background

Carotid stenosis is characterized by progressive narrowing of the carotid arteries, most commonly due to atherosclerotic plaque formation, which leads to compromised cerebral perfusion and an increased risk of ischemic cerebrovascular events [1, 2]. The pathological process involves endothelial dysfunction, lipid accumulation, and inflammatory cell infiltration, which may result in luminal obstruction and potential plaque rupture [3]. As a major etiological factor in ischemic stroke, carotid stenosis represents a critical target for both preventive and therapeutic interventions [2, 4]. Surgical revascularization procedures, particularly carotid endarterectomy, are performed to reduce the risk of cerebral ischemia by restoring adequate blood flow to the brain [5, 6].

The carotid artery primarily supplies ocular blood flow and may be affected by carotid stenosis and surgical interventions, thereby influencing visual function [7, 8]. Electrophysiological assessment of the eye, particularly through techniques such as electroretinography (ERG) and visual evoked potentials (VEP), provides an objective measure of retinal and post-retinal visual pathway function that is highly sensitive to ischemia [9, 10]. In the context of high-grade carotid stenosis, reduced perfusion of the ophthalmic and central retinal circulation, as well as distal microembolization, can lead to retinal and optic nerve ischemia, which manifests as reductions in ERG amplitudes, prolongation of implicit times, and delayed or diminished VEP responses. These electrophysiological alterations reflect dysfunction of photoreceptors, bipolar cells, and ganglion cells. They may precede overt structural or fundoscopic changes, thereby linking carotid-related ocular hypoperfusion to measurable disturbances in visual electrophysiology [11].

The objective of this study is to systematically synthesize and evaluate the evidence on visual electrophysiology in patients undergoing carotid endarterectomy, focusing on how electroretinography and visual evoked potentials capture ischemia-related dysfunction of the visual pathways and their recovery after revascularization, to clarify their clinical value for assessing ocular and neuroretinal outcomes in carotid stenosis.

## Methods

### Protocol and reporting framework

This systematic review was conducted in accordance with the Preferred Reporting Items for a Systematic Review and Meta-analysis (PRISMA) Statement [12]. Institutional review board approval was not obtained due to the nature of this study. The review protocol has been registered with Prospero (CRD420251129351).

### Search strategy

We systematically searched MEDLINE (via PubMed), Scopus, and Web of Science from inception to August 2025. No language, country, or publication status restrictions were applied. The search strategy combined controlled vocabulary and free-text terms for carotid endarterectomy, carotid stenosis, and ocular electrophysiology exams (Supplemental Table 1). Additional studies were identified by screening the reference lists of included articles and relevant reviews, conducting forward/backward citation tracking, and corresponding with field experts.

### Selection criteria

We included peer-reviewed studies enrolling adults (≥ 18 years) with carotid artery stenosis undergoing carotid endarterectomy, in whom retinal or optic nerve electrophysiology was assessed using electroretinography (full-field, pattern, or multifocal), visual evoked potentials, electro-oculography, or other multifocal electrophysiologic recordings before and after the surgical intervention. Randomized, quasi-randomized, cohort, case-control, and cross-sectional designs were eligible, as were large case-series (≥ 20 participants) with pre-, post-, or comparator data.

We excluded pediatric studies; hereditary dystrophies without a vascular sub-analysis; animal or in-vitro studies; intraoperative monitoring only; case reports or case series with < 20 patients; conference abstracts without full text; and studies without electrophysiology.

### Study selection and data extraction

Records were deduplicated and screened in two stages (titles/abstracts, then full text) by two independent reviewers (S.P.M. and A.F.) using a dedicated screening platform. Disagreements were resolved by discussion or third-reviewer adjudication (J.R.N).

Two reviewers independently extracted study-level data using a piloted form: study design, sample characteristics, carotid disease context, electrophysiological modality and parameters, timing relative to surgery, comparators, structural or functional co-outcomes, effect metrics, and follow-up duration. The study authors were contacted to obtain missing or unclear information.

### Assessment of study quality

Risk of bias was independently assessed by two reviewers using the NHLBI Study Quality Assessment Tools appropriate to the study design (at https://www.nhlbi.nih.gov/health-topics/study-quality-assessment-tools - Accessed in 11/2025).

The quality of evidence of the articles included in the systematic review was evaluated using the Grading of Recommendations, Assessment, Development, and Evaluation (GRADE) [13].

### Outcomes

Primary outcomes are post-operative changes in ocular electrophysiological parameters: VEP amplitudes and latency; ERG amplitudes, latency and oscillatory potential amplitudes. Secondary outcomes include visual acuity, intraocular pressure, optical coherence tomography (OCT), and perimetry parameters.

### Quantitative synthesis

Quantitative synthesis was planned if studies were sufficiently homogeneous in design, patient populations, and electrophysiological endpoints. As a meta-analysis was not appropriate due to heterogeneity, a structured narrative synthesis was performed.

## Results

### Search results

**Fig. 1:**
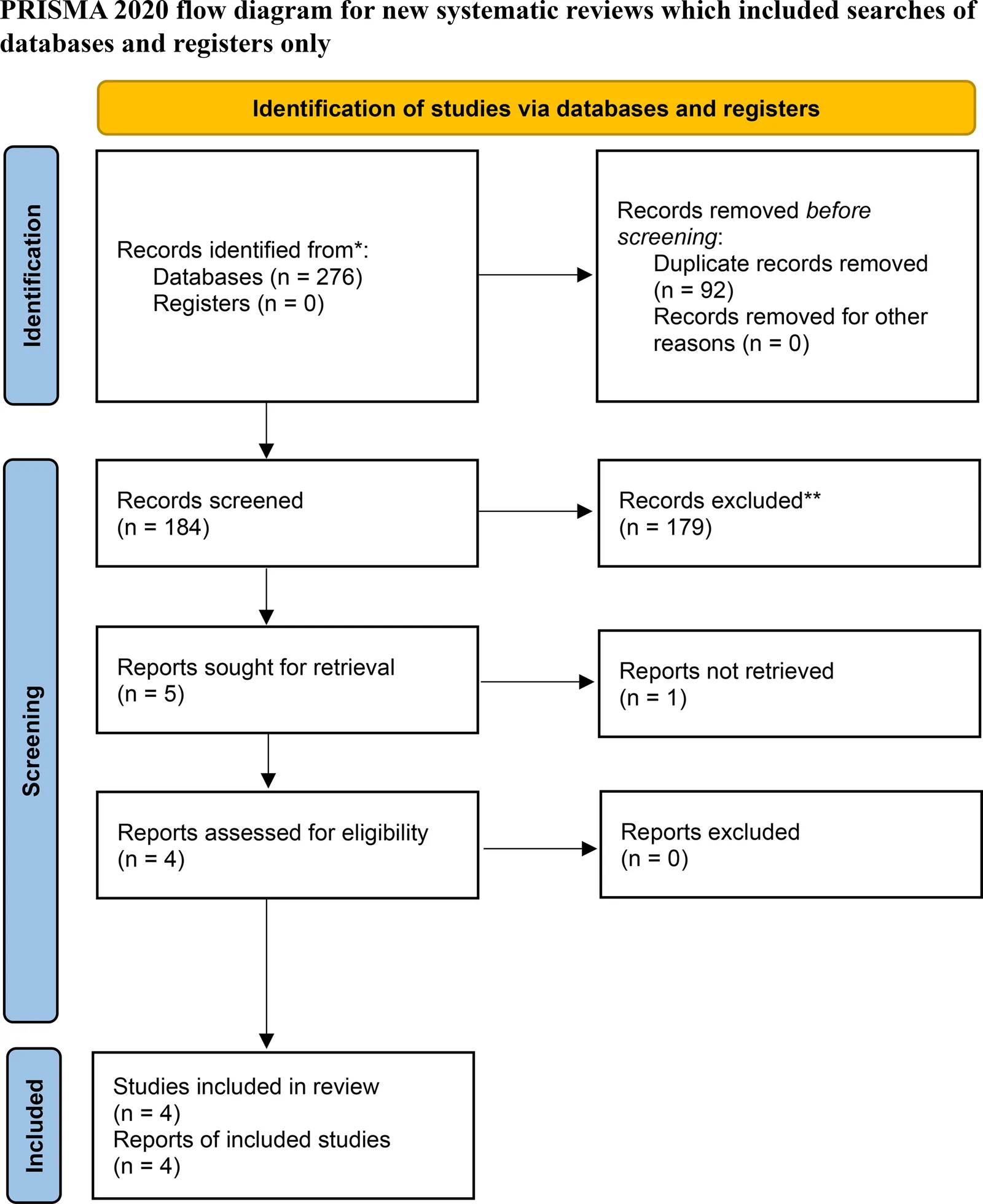
Flow-diagram according to PRISMA statement regarding the process of identification and selection of the studies *Consider, if feasible to do so, reporting the number of records identified from each database or register searched (rather than the total number across all databases/registers). **If automation tools were used, indicate how many records were excluded by a human and how many were excluded by automation tools

After the database search and duplicate exclusion, 184 studies were screened. After selection based on title and abstract, 179 studies were excluded. 5 studies were eligible for full-text assessment; none were excluded, but 1 could not be retrieved (Fig. 1). Thus, a total of 4 published articles were included in this systematic review [14–17].

### Description of studies

A total of 4 prospective observational studies were included in the final qualitative synthesis, comprising 95 patients who underwent carotid endarterectomy (CEA). The studies were published between 1998 and 2023 and originated from Europe (Poland and Italy) and Asia (China), reflecting a geographically heterogeneous but limited evidence base. Sample sizes were small, ranging from 12 to 46 patients, and all investigations were conducted at a single academic center. Follow-up duration varied across studies, ranging from postoperative assessment only, without a clearly defined follow-up duration [14] to 9 months [15]. Incomplete reporting of methodological and outcome details was identified in one study [15]. Main characteristics of the included studies are presented in Table 1. No randomized controlled trials were identified. Demographic and comorbidity data for the populations included in the studies were collected and are presented in Table 2.

**Table 1:** Main characteristics of the included studies

Author	Year	Country	Journal	Study Design	Study Center	Preoperative assessment	Postoperative assessment	Sample Size (patients)	No CEA	GRADE Evaluation
*Krasińska-Plachta et al.*[15]	2023	Poland	Polish Journal of Surgery	Prospective observational	Poznan University of Medical Sciences	Before CEA (timing not specified)	Early postoperative period (timing not specified)	22	22	Low
*Yan et al.*[17]	2019	China	Annals of Vascular Surgery	Prospective observational	QingdaoUniversity	Before CEA (timing not specified)	4 months after CEA	15	15	Low
*Machalińska et al.*[18]	2017	Poland	Polish archives of internal medicine	Prospective observational	Pomeranian Medical University	Before CEA (timing not specified)	3 months after CEA	46	46	Low
*Brovarone et al.*[16]	1998	Italy	Acta ophthalmologica scandinavica	Prospective observational	University of Turin	Before CEA (timing not specified)	9 months after CEA	12	12	Low

NR - Not reported; CEA - Carotid endarterectomy; GRADE - Grading of Recommendations, Assessment, Development, and Evaluation

**Table 2:** Participants demographic and comorbidity data

Author	No patients (no eyes)	Age (years), mean ± SD	Male	AHT	Dyslipidemia	Statins	DM	BMI (Kg/m^2^), mean	Smoking History	CAD	PAD	HF
*Krasińska-Plachta et al.*[15]	22 (44)	68.7 ± 9.5	11 (50%)	18 (82%)	NR	NR	8 (37%)	NR	5 (23%)	NR	NR	NR
*Yan et al.*[17]	15 (30)	62.8 ± 5.0	11 (74%)	10 (67%)	NR	NR	9 (60%)	NR	NR	5 (34%)	NR	NR
*Machalińska et al.*[18]	46 (92)	63.5 ± 6.1	27 (59%)	38 (83%)	NR	NR	NR	27.8	42 (91%)	16 (35%)	12 (26%)	NR
*Brovarone et al.*[16]	12 (24)	67.8 ± 7.8	NR	NR	NR	NR	NR	NR	NR	NR	NR	NR

NR - Not reported; SD - Standard Deviation; AHT - Arterial Hypertension; DM - Diabetes Mellitus; BMI - Body Mass Index; CAD - Coronary Artery Disease; PAD - Peripheral Artery Disease; HF - Heart Failure

Mean age was consistently in the sixth to seventh decade, ranging from 62.8 ± 5.0 to 68.7 ± 9.5 years. Where reported, the proportion of male participants ranged from 50% to 74% [14, 16]. Arterial hypertension was highly prevalent, affecting approximately 67–83% of patients in studies reporting this variable. Diabetes mellitus was reported in two studies, with prevalence ranging from 37% to 60%. Three studies excluded patients with severe ocular diseases or other conditions potentially affecting the retina or optic nerve [14, 16, 17]. Data on dyslipidemia, statin use, and heart failure were not reported across studies. Among vascular comorbidities, coronary artery disease was present in up to 35% of patients in the studies that reported these data [16, 17], while peripheral artery disease was reported in 26% of participants in one study [17]. Smoking history was reported in one study, with a prevalence of 91% [17].

All included studies enrolled patients with severe carotid stenosis ≥ 70%, except one that used a ≥ 60% threshold, which remains consistent with current European Society for Vascular Surgery (ESVS) guideline recommendations [4, 14]. Reporting of symptom status was inconsistent, with detailed clinical descriptions available in only one study [16], which documented a heterogeneous spectrum of neurological presentations, including focal limb weakness, dizziness, hypersomnia, and symptoms suggestive of cerebral infarction, alongside a small proportion of asymptomatic patients. Data on amaurosis fugax and on the formal classification of symptomatic versus asymptomatic carotid stenosis were largely not reported. Perioperative medical management was insufficiently described across most studies. However, in one cohort [17], all patients received preoperative single antiplatelet therapy with acetylsalicylic acid, and systemic intraoperative heparinization was universally administered. Information regarding dual antiplatelet therapy, chronic anticoagulation, and anesthetic technique was sparse, with local anesthesia explicitly reported in only one study [17]. The characteristics of included participants described above are presented in Table 3.

**Table 3:** Characteristics of included participants - Carotid stenosis severity, symptoms, and perioperative management

Author	Carotid Stenosis Mean (%)		Amaurosis Fugax	Symptomatic Carotid Stenosis	SAPT (%)	DAPT (%)	Anticoagulation^1^ (%)	Anesthesia Type
	Ipsilateral	Contralateral						
*Krasińska-Plachta et al.* [15]	> 60	NR	NR	0	NR	NR	NR	NR
*Yan et al.* [17]	> 70	NR	0	weakness in certain limbs (*n* = 6), dizziness or hypersomnia (*n* = 3), suspected cerebral infarction with symptoms of clumsiness or numbness around the mouth (*n* = 3), asymptomatic (*n* = 3)	NR	NR	NR	NR
*Machalińska et al.* [18]	≥ 70	NR	0	0	100^2^	0	100	Local
*Brovarone et al.* [16]	> 80	NR	NR	NR	NR	NR	NR	NR

SAPT - Single antiplatelet therapy; DAPT - Dual antiplatelet therapy; NR - Not reported

^1^ - Systemic Heparinization during surgery

^2^ - The patients were preoperatively treated with oral acetylsalicylic acid at a dose of 75 mg/d for at least 10 days prior to CEA

### Main findings

Structural, visual, and electrophysiological outcomes before and following carotid endarterectomy are presented in Table 4. Notably, substantial heterogeneity in outcome definitions and reporting was observed. Electrophysiological assessments revealed a divergent pattern between ERG and VEP findings. ERG parameters, particularly oscillatory potential amplitudes and a-/b-wave amplitudes, demonstrated postoperative improvement in three of the four included studies [14, 16, 17], suggesting a degree of retinal functional recovery following restoration of carotid blood flow. In contrast, pattern and flash visual evoked potentials (PVEP/VEP) showed no consistent postoperative changes in amplitude or latency across the three studies that assessed them [17] with findings that were largely heterogeneous and inconclusive. This dissociation between ERG improvement and the absence of consistent VEP changes suggests that postoperative functional recovery may be predominantly confined to the retinal level, with less evidence of recovery along the post-retinal visual pathways. Retinal nerve fiber layer (RNFL) thickness showed no significant postoperative change across studies that evaluated it using OCT [14, 16, 17]. In contrast, visual acuity and both static and kinetic visual field parameters demonstrated postoperative improvement [16]. Changes in intraocular pressure (IOP) were reported inconsistently: a quantitative reduction was documented in one study [16], no change was found by Krasinska-Plachta et al. [14], and Brovarone et al. [15] reported that 70% had unchanged IOP after surgery, with the remaining 30% demonstrating a decrease. Overall, the diversity of testing modalities, outcome parameters, and reporting formats precluded meaningful quantitative pooling of most endpoints and limited the interpretability of between-study comparisons.

**Table 4:** Summary of reported functional and structural outcomes

Author	Test	Outcome Parameter	Change	Statistical Significance (*P*-value)	Follow-up
*Krasińska-Plachta et al.* [15]	Pattern VEP	Amplitude and latency	No change	NR	Early postoperative interval
	Visual Fields	Mean deviation	Improvement	< 0.05	
	IOP	Intraocular pressure	No change	NR	
	OCT	RNFL thickness	No change	NR	
*Yan et al.* [17]	Visual Acuity	Uncorrected and BCVA	Improvement	< 0.05	4 Months
	IOP	Intraocular pressure	Decrease	0.0022	
	Static Visual Fields	Mean sensitivity	Improvement	0.0208	
		Mean deviation	Improvement	0.025	
	Kinetic Visual Fields	Range	Improvement	0.0126	
	OCT	RNFL thickness	No change	> 0.05	
	Flash VEP	Latency	Improvement	0.0151	
	Pattern VEP	Amplitude and latency	No change	> 0.05	
	ERG	Oscillatory potential amplitudes	Improvement	< 0.001	
*Machalińska et al.* [18]	OCT	RNFL thickness	No change	> 0.05	3 Months
	Pattern ERG	Amplitude and latency	No change	> 0.05	
	Pattern VEP	Amplitude and latency	No change	> 0.05	
	Full-field ERG	Multiple wave amplitudes	Improvement	< 0.05	
*Brovarone et al.* [16]	Visual Acuity	Visual Acuity	No change	NR	Follow-up extended to 9 months
	IOP	Intraocular pressure	70% no change 30% decrease	NR	
	Visual Fields	Multiple parameters	No consistent change	NR	
	VEP	Amplitude and latency	Improvement	NR	
	Pattern ERG	Amplitude and latency	Improvement	NR	

VEP - Visual Evoked Potentials; IOP - Intraocular Pressure; OCT - Optical Coherent Tomography; RNFL - Retinal Nerve Fiber Layer; BCVA - Best Corrected Visual Acuity; ERG - Electroretinogram; CEA - Carotid Endarterectomy; NR - Not Reported

### Studies quality

**Fig. 2:**
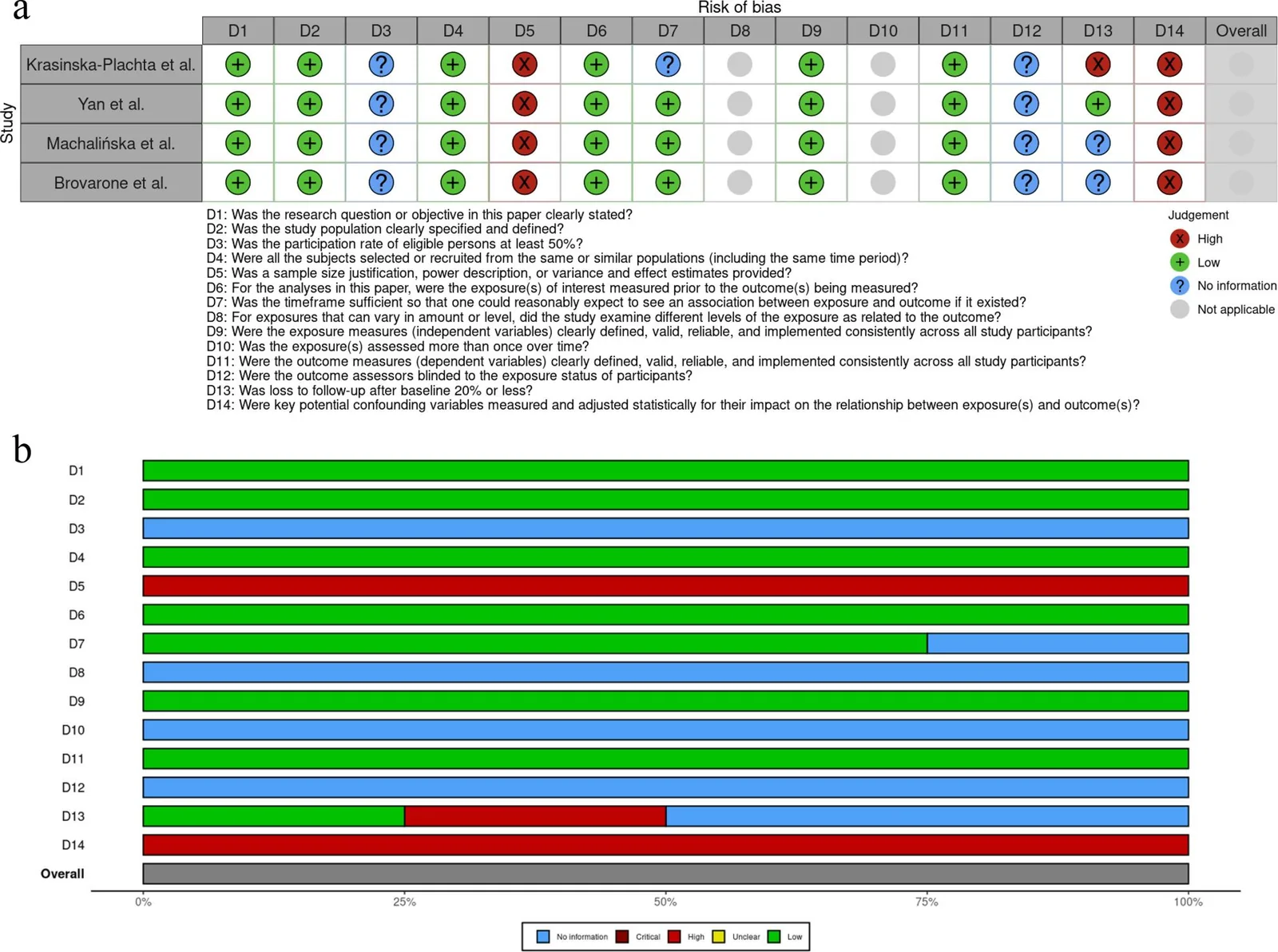
(**a**) Risk of bias assessment for all studies included in the systematic review, presented by individual article; (**b**) Risk of bias assessment for all included studies, presented by individual item

The risk of bias for each observational cohort is individually displayed in Fig. 2a, whereas the overall judgement per evaluated item regarding observational cohorts is shown in Fig. 2b.

Overall risk of bias was moderate to high, driven primarily by consistently high risk across key methodological domains. All included studies lacked justification for sample size/power or precision considerations (D5) and did not adequately measure and statistically adjust for important confounders (D14). Most studies were judged to be at low risk for having a clearly stated objective (D1), well-defined populations (D2), comparable participant selection (D4), exposure assessed before outcomes (D6), clearly defined exposure measures (D9), and clearly defined outcome measures (D11).

Importantly, none of the included studies reported adjusted statistical models. Therefore, all reported associations are derived from unadjusted analyses and may be influenced by residual confounding from factors such as age, diabetes mellitus, hypertension, smoking status, baseline ocular status, and severity of carotid stenosis.

### Publication bias

Due to substantial clinical and methodological heterogeneity and inconsistent reporting of outcomes across studies, quantitative data synthesis was not feasible. Consequently, assessment of publication bias using funnel plots or Egger’s regression test was not performed.

## Discussion

Carotid endarterectomy aims to restore normal cerebral perfusion and reduce stroke risk; however, by improving inflow through the internal carotid artery, it may also alter ocular circulation, potentially affecting retinal and choroidal blood flow [18–20]. The data synthesized in this review suggest a discordant pattern between structural and functional outcomes, with no postoperative changes observed in RNFL thickness. In contrast, functional visual measures and electroretinographic outcomes showed improvement in the four studies [14–17]. Collectively, these findings suggest functional responsiveness of the visual system after carotid revascularization, while underscoring the need for standardized methodologies and higher-quality prospective studies to clarify the clinical relevance and mechanisms underlying these observations.

Among the outcomes assessed, electrophysiological parameters — particularly ERG-derived measures — showed the most frequent postoperative changes across included studies, though the limited and heterogeneous evidence base precludes definitive conclusions regarding their sensitivity or diagnostic value. Improvements were most consistently observed in electroretinographic parameters, particularly amplitude-related measures, suggesting enhanced retinal and visual pathway function after restoration of carotid blood flow [15–17]. Given the high metabolic demand of retinal neurons and their susceptibility to chronic hypoperfusion [18, 21], these findings support the hypothesis that carotid stenosis may induce a functional impairment that is, at least, partially reversible. In contrast, changes in visual evoked potentials were less consistent across studies, indicating that electrophysiological recovery after revascularization may predominantly reflect improved neuronal responsiveness rather than uniform recovery along the entire visual pathway [10, 14]. Overall, these preliminary findings suggest that ocular electrophysiology, particularly ERG, may capture functional changes following carotid revascularization, though the clinical relevance of these changes requires confirmation in adequately designed prospective studies. ERG improvements, most consistently involving oscillatory potentials and a-/b-wave amplitudes, were observed in three of the four included studies, suggesting that retinal function, given its high metabolic demand and susceptibility to chronic hypoperfusion, may exhibit at least partial functional recovery following restoration of carotid blood flow [15–17]. This pattern is biologically plausible, as the retina is directly supplied by the ophthalmic artery and is therefore among the first structures to benefit from improved perfusion after revascularization. In contrast, VEP parameters showed no consistent postoperative changes across the three studies that assessed them, with findings that were heterogeneous and largely inconclusive [14, 16, 17]. This dissociation is clinically important: it suggests that while retinal function may recover following revascularization, the post-retinal visual pathways may be less responsive, possibly reflecting more established or irreversible ischemic injury at those levels, or alternatively, the greater susceptibility of VEP to confounding factors such as cortical state, attention, and inter-individual variability [8, 22]. These observations reinforce the specificity of ERG over VEP as a marker of perioperative retinal functional change in the context of carotid disease, though this conclusion remains preliminary given the limited evidence base [8–10, 22].

In addition to electrophysiological outcomes, functional visual measures assessed by perimetry demonstrated postoperative improvement in two cohorts [14, 16]. Improvements in visual field indices, including mean deviation, sensitivity, and kinetic field extent, are clinically meaningful, as they reflect integrated retinal and post-retinal function and are directly relevant to patients’ visual performance. The concordance between electrophysiological improvement and visual field changes supports the functional significance of the observed neurophysiological recovery [22]. However, visual field outcomes were inconsistently reported, and in some studies, results were heterogeneous, limiting direct comparisons. These findings nevertheless suggest that functional gains detected with electrophysiology may translate, at least in part, into measurable improvements in visual perception.

In contrast to functional outcomes, structural parameters remained largely unchanged after carotid endarterectomy. RNFL thickness showed no consistent postoperative variation, reinforcing the concept that revascularization does not reverse established anatomical damage [23]. This dissociation between functional improvement and structural stability supports the notion that electrophysiological alterations may precede detectable structural loss on optical coherence tomography [7]. Similarly, intraocular pressure did not demonstrate consistent postoperative changes [14–16]. The mechanisms underlying potential variation in IOP remain poorly understood and may reflect indirect hemodynamic effects rather than a direct consequence of carotid revascularization [24, 25]. Taken together, these findings emphasize that the benefits of carotid endarterectomy on visual outcomes are predominantly functional and physiological rather than structural.

Several limitations of this systematic review should be acknowledged. First, only four eligible studies comprising 95 patients met the inclusion criteria despite a comprehensive search strategy across three major databases and more than two decades of published literature. Most of the available evidence is based on small observational cohorts, frequently without formal sample-size justification or power calculations, which limits the precision, robustness, and generalizability of the reported findings. In addition, there was substantial heterogeneity across studies regarding baseline patient characteristics, symptom status, degree of carotid stenosis, perioperative management, electrophysiological protocols, and follow-up duration. The absence of randomized controlled trials, inconsistent reporting of clinical variables, and variability in outcome definitions and testing modalities precluded meaningful quantitative synthesis and limit causal inference. Furthermore, the limited number of included studies precluded advanced analyses, such as multivariable meta-regression, and restricted the assessment of associations between electrophysiological outcomes and other short- and long-term clinical endpoints.

Despite these limitations, the findings of this systematic review highlight the potential role of ocular electrophysiology, particularly electroretinography, as a sensitive and objective tool for detecting functional impairment of the visual pathways associated with carotid stenosis and for assessing the response to surgical revascularization. The observed functional improvements in the absence of structural recovery suggest that electrophysiological testing may identify reversible ischemic dysfunction before irreversible anatomical damage occurs. These results underscore the need for larger prospective cohorts and well-designed randomized controlled trials with standardized electrophysiological protocols and clearly defined outcomes to validate current evidence and address existing knowledge gaps. Improved understanding of the impact of carotid revascularization on visual function may ultimately inform clinical decision-making, patient selection, and postoperative monitoring strategies, aiming to optimize visual and neurological outcomes while minimizing potential long-term harm.

## Conclusion

This systematic review suggests that carotid endarterectomy may be associated with electrophysiological changes, particularly in ERG-derived parameters. Electroretinographic parameters showed the most consistent postoperative changes across studies, although the limited and heterogeneous evidence base precludes definitive conclusions regarding their clinical significance. In contrast, VEP showed heterogeneous findings, whereas structural parameters, such as retinal nerve fiber layer thickness, remained largely unchanged. However, the available evidence remains preliminary and exploratory. These findings should be interpreted with caution due to the limited number of studies, small sample sizes, and substantial heterogeneity in study designs, methodologies, and outcome assessments. Nevertheless, the available evidence is insufficient to establish ocular electrophysiology as a validated tool in this context, but provides a preliminary rationale for larger, methodologically rigorous prospective studies. Further well-designed prospective studies with standardized electrophysiological protocols are required to confirm these findings and clarify their clinical relevance.

## Supplementary Information

Below is the link to the electronic supplementary material.

Supplementary Material 1

## Data Availability

The data supporting the findings of this study are available from the corresponding author upon reasonable request.

## Abbreviations

CEA: Carotid Endarterectomy
ERG: Electroretinography
EOG: Electrooculography
IOP: Intraocular Pressure
OCT: Optical Coherence Tomography
PVEP: Pattern Visual Evoked Potentials
RNFL: Retinal Nerve Fiber Layer
VEP: Visual Evoked Potentials
