# Supplementary material for: Visual electrophysiology in carotid endarterectomy: a systematic review
Source: Int J Retina Vitreous. 2026 Jun 29;12:128. doi: 10.1186/s40942-026-00895-2 (PMC13584395; doi:10.1186/s40942-026-00895-2)
Supplement: Supplementary file 1 [file 40942_2026_895_MOESM1_ESM.docx]

**Supplemental Table 1** - Search query

| **Bibliographic source** | **Search term** |
| --- | --- |
| **Pubmed** | ("Carotid Stenosis"[MeSH Terms] OR "Carotid Artery Diseases"[MeSH Terms] OR "Carotid Stenosis"[Title/Abstract] OR "Carotid Artery Disease"[Title/Abstract] OR "Endarterectomy, Carotid"[MeSH Terms] OR "carotid endarterectomy"[Title/Abstract] OR "CEA"[Title/Abstract])  AND  ("Electroretinography"[MeSH Terms] OR "Electroretinogram"[Title/Abstract] OR "ERG"[Title/Abstract] OR "Pattern electroretinogram"[Title/Abstract] OR "pattern ERG"[Title/Abstract] OR "PERG"[Title/Abstract] OR "multifocal ERG"[Title/Abstract] OR "mfERG"[Title/Abstract] OR "multifocal electroretinogram"[Title/Abstract] OR "Evoked Potentials, Visual"[MeSH Terms] OR "Visual Evoked Potentials"[Title/Abstract] OR "Visual Evoked Potential"[Title/Abstract] OR "VEP"[Title/Abstract] OR "pattern VEP"[Title/Abstract] OR "flash VEP"[Title/Abstract] OR "PVEP"[Title/Abstract] OR "Electrooculography"[MeSH Terms] OR "electro-oculography"[Title/Abstract] OR "electrooculography"[Title/Abstract] OR "EOG"[Title/Abstract]) |
| **SCOPUS** | TITLE-ABS-KEY("carotid stenosis" OR "carotid artery disease*" OR "carotid endarterectomy" OR "CEA")  AND  TITLE-ABS-KEY("electroretinogra*" OR "ERG" OR "pattern ERG" OR "PERG" OR "multifocal ERG" OR "mfERG" OR "visual evoked potential*" OR "VEP" OR "pattern VEP" OR "flash VEP" OR "PVEP" OR "electrooculogra*" OR "electro-oculogra*" OR "EOG") |
| **Web of science** | TS=("carotid stenosis" OR "carotid artery disease*" OR "carotid endarterectomy" OR "CEA")  AND  TS=("electroretinogra*" OR "ERG" OR "pattern ERG" OR "PERG" OR "multifocal ERG" OR "mfERG" OR "visual evoked potential*" OR "VEP" OR "pattern VEP" OR "flash VEP" OR "PVEP" OR "electrooculogra*" OR "electro-oculogra*" OR "EOG") |

**Supplemental Table 2** - Covariates used in the adjusted models

| **Author** | **Covariables used in adjusted models** |
| --- | --- |
| *Krasinska-Plachta et al.* [15] | NR |
| *Yan et al.* [17] | NR |
| *Machalińska et al.* [18] | NR |
| *Brovarone et al.* [16] | NR |

NR – Not Reported
